# Retraction Note: Optical rectification and absorption coefficients studied by a short-range topless exponential potential well with inverse square root

**DOI:** 10.1038/s41598-020-65240-5

**Published:** 2020-05-15

**Authors:** Qiucheng Yu, Kangxian Guo, Meilin Hu

**Affiliations:** 1 Department of Physics, School of Physics and Electronic Engineering, Guangzhou University, Guangzhou, 510006 China; 2 Guangdong Provincial Engineering and Technology Research Center of Semiconductor Lighting and Backlighting, Guangzhou, 510006 China; 3 Dipartimento di Fisica, Universitàdi Napoli Federico II, Complesso Universitario di Monte Sant’Angelo, via Cintia, Napoli, 80126 Italy

Retraction of: *Scientific Reports* 10.1038/s41598-019-38519-5, published online 19 February 2019

The authors are retracting this Article.

There is an error in the derivation described in the Article. Function z(x) as defined in the Article does not fulfil Eq. (5). This makes subsequent steps of the derivation incorrect. Additionally, the Article also states that Eq. (8) is a solution to Eq. (4). This too is incorrect. Overall, the authors are not able to guarantee the results and conclusions, and decided to retract the Article.

All authors agree to retraction.

